# Nosology of Juvenile Muscular Atrophy of Distal Upper Extremity: From Monomelic Amyotrophy to Hirayama Disease—Indian Perspective

**DOI:** 10.1155/2013/478516

**Published:** 2013-08-26

**Authors:** Kaukab Maqbool Hassan, Hirdesh Sahni

**Affiliations:** ^1^Division of Neurology, Department of Internal Medicine, Command Hospital, Alipore, West Bengal University of Health Sciences, Kolkata 700 027, India; ^2^Department of Neuroradiology & Interventional Radiology, Command Hospital (Air Force), Bangalore 560007, India

## Abstract

Since its original description by Keizo Hirayama in 1959, “juvenile muscular atrophy of the unilateral upper extremity” has been described under many nomenclatures from the east. Hirayama disease (HD), also interchangeably referred to as monomelic amyotrophy, has been more frequently recognised in the west only in the last two decades. HD presents in adolescence and young adulthood with insidious onset unilateral or bilateral asymmetric atrophy of hand and forearm with sparing of brachioradialis giving the characteristic appearance of oblique amyotrophy. Symmetrically bilateral disease has also been recognized. Believed to be a cervical flexion myelopathy, HD differs from motor neuron diseases because of its nonprogressive course and pathologic findings of chronic microcirculatory changes in the lower cervical cord. Electromyography shows features of acute and/or chronic denervation in C7, C8, and T1 myotomes in clinically affected limb and sometimes also in clinically unaffected contralateral limb. Dynamic forward displacement of dura in flexion causes asymmetric flattening of lower cervical cord. While dynamic contrast magnetic resonance imaging is diagnostic, routine study has high predictive value. There is a need to lump all the nomenclatures under the rubric of HD as prognosis in this condition is benign and prompt diagnosis is important to institute early collar therapy.

## 1. Introduction

Muscular atrophy of distal upper extremity in young males has been well described from Asia, particularly Japan and India, over the last half a century [1–5]. Though the entity has been less well recognized in North America and Europe, there have been case reports and short case series in the last two decades [6–10]. The entity has been described by various authors under different nomenclatures.

Hirayama et al. from Japan first reported in 1959 the entity of “juvenile muscular atrophy of the unilateral upper extremity” [11]. This entity, now increasingly referred to as Hirayama disease (HD), is a rare disease affecting primarily young men in the second to third decades of life [12]. Since then, similar patients in their adolescence and young adulthood have been described under a plethora of names not only in Japan but also in other Asian countries as well as in Europe and North America [13]. Biondi et al. called it juvenile muscular atrophy of the distal upper extremity (JMADUE) [6]. Gourie-Devi et al. in 1984 reported monomelic amyotrophy (MMA) from South India [4]. Pradhan and Gupta in their report in 1997 called this condition “juvenile asymmetric segmental spinal muscular atrophy” (JASSMA) [14]. 

HD is characterized by the insidious onset of unilateral or asymmetric atrophy of the hand and forearm, with sparing of brachioradialis giving the characteristic appearance of oblique amyotrophy [15]. It is thought to be a cervical myelopathy related to flexion movements of the neck [3]. HD differs from classic types of motor neuron diseases (MNDs) because of its nonprogressive course and pathologic findings of chronic microcirculatory changes in the territory of the anterior spinal artery supplying anterior horns of the lower cervical cord [1, 12, 16]. The pathogenetic mechanism of this disease is attributed to forward displacement of the posterior wall of the lower cervical dural canal when the neck is in flexion, which causes marked, often asymmetric, flattening of the lower cervical cord [1, 2, 12, 17]. 

A spectrum of diagnostic magnetic resonance imaging (MRI) features has been described in the literature [5, 6, 14, 18–20]. HD should be more widely recognized as prognosis in this condition is benign, and early detection and effective treatments may be considered [13].

## 2. Clinical Features

HD is a benign disorder with a stationary stage after a progressive course for up to six or fewer years. It occurs mainly in young males between the ages of 15 and 25 years [1, 13]. The characteristic clinical features as originally described by Hirayama include (a) weakness and wasting predominantly in C7, C8, and T1 myotomes in one upper limb or asymmetrically in both upper limbs, (b) insidious onset in teens or early twenties, (c) progression for one to three years followed by arrest of disease or relatively benign course, (d) irregular coarse tremors (minipolymyoclonus) in the fingers of the affected hand(s), (e) mild transient worsening of symptoms on exposure to cold, (f) electromyography evidence of chronic denervation in the clinically or subclinically affected muscles, and (g) absence of substantial sensory loss/reflex abnormalities, cranial nerve, pyramidal tract in lower limb, sphincter, or cerebellar deficits [12].

Gourie-Devi et al. in 1984 reported thirteen cases of single upper limb atrophy from South India, which she called MMA. The characteristic clinical features were insidious onset in the second and third decades, male preponderance, sporadic occurrence, wasting and weakness confined to one limb, and absence of involvement of the cranial nerves, cerebrum, brain stem, and sensory system. They observed slow progression of illness for two to four years followed by a stationary phase. There was no clinical evidence of involvement of the other three limbs even in patients with long-standing illness of ten- to fifteen-year duration [4]. 

In a later study in 2003, Gourie-Devi and Nalini reported on followup of forty-four patients of single upper limb atrophy with duration of illness of 5 years or more. They found progression of the disease in 79.5% patients up to 5 years and in 4.6% patients up to 8 years; subsequent to attaining a stationary course, none of the 44 subjects developed fresh symptoms or signs during a mean follow-up period of 9.7 years (range 2.5–23 years). Seven patients (15.9%) at presentation had minimal involvement of contralateral upper limb with gross asymmetry, and later one more patient developed similar features. Spread to the contralateral upper limb with minimal neurologic deficit was seen in less than 20% of patients, but involvement of lower limbs was not observed. MMA did not evolve to amyotrophic lateral sclerosis. None of the patients developed involvement of cranial nerves, lower limbs, or pyramidal signs [21].

Pradhan and Gupta reported in 2001 from North India thirty-five patients with juvenile asymmetric upper limb wasting seen between 1993 and 2000 [22]. In a later report in 2009, Pradhan reported bilaterally symmetric involvement in 10% of 106 patients with HD seen between 1992 and 2008. Nine of these had unilateral onset. The important characteristics of this type of presentation included severe weakness and wasting in C7, C8, and T1 myotomes that frequently spilled over to C6 segment, predominant autonomic dysfunction in distal upper extremities in the form of cold paresis, cold skin, excessive sweating, and hair loss over the dorsum of the hands, and a very prominent bilateral minipolymyoclonus [23].

There have been three well-reported series from India over the last five years (Table 1). Hassan et al. in 2012 have reported eleven cases of HD from various regions of India, while Sonwalkar et al. in 2008 reported eight cases from South India and Raval et al. in 2010 reported nine cases from North India [5, 18, 24]. All patients in these series were males. The duration of illness at the time of presentation varied from 3 months to 36 months with large majority presenting in the first 2 years of onset [5, 18]. 

Sonwalkar et al. reported a mean age of 18.5 years (11–24 years) [18], while Raval et al. reported a mean age of 18 years (15–23 years) [24]. Hassan et al. reported all patients younger than 25 years (median age 23 years; 19–50 years) except one patient who was 50 years old with disease duration of 2 years. The authors postulated that the patient with late-onset disease perhaps had milder disease in his youth, with secondary worsening later in life with MRI findings consistent with HD [5].

**Figure 1:**
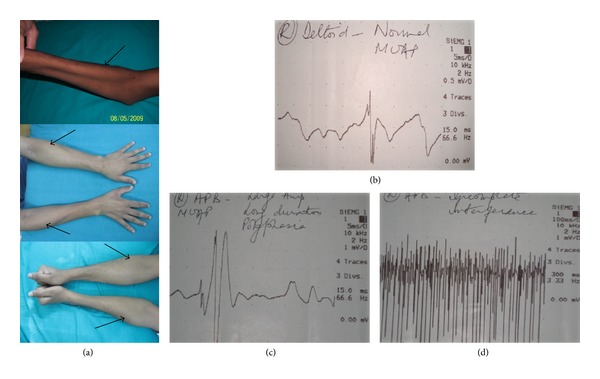
Wasting of hands and forearms with oblique amyotrophy (black arrows) (a); electromyography showing normal motor unit action potential in right deltoid (b); neurogenic motor unit action potential in right abductor pollicis brevis (c); incomplete interference pattern on submaximal volition in right abductor pollicis brevis (d).

These authors reported clinical features characterized by insidious onset, predominantly unilateral upper extremity weakness and atrophy, cold paresis, and no sensory or pyramidal tract involvement [5, 18, 24]. The amyotrophy is unilateral in most patients, asymmetrically bilateral in some, and rarely symmetric in others [23, 24]. Raval et al. found unilateral findings in 89% while Sonwalkar et al. and Hassan et al. found bilateral involvement in the majority of cases (75% and 55%, resp.) [5, 24]. Hassan et al. found oblique amyotrophy or sparing of brachioradialis in all their patients (Figure 1(a)), while Sonwalkar et al. reported this in 37% and Raval et al. in 67.5% cases [5, 18, 24]. Hassan et al. and Sonwalkar et al. found hand weakness in all their patients [5, 18]. Hassan et al. found hand and forearm wasting in all their patients, while Sonwalkar et al. reported this in 75% and 50%, respectively [5, 18]. Hassan et al. found minipolymyoclonus in 91% of their patients, while Sonwalkar et al. reported this in 62% and Raval et al. in 55% cases [5, 18, 24]. Hassan et al. reported fasciculations in 27% of their patients, while Raval et al. reported this in 55% cases [5, 24]. Hassan et al. found lower limb hyperreflexia in 18% of their patients, while Sonwalkar et al. reported this in 12% cases [5, 18]. Hassan et al. reported cold paresis in 36% of their patients, while Raval et al. reported this in 55% cases [5, 24]. Dysaesthesia of hand has been reported in 9–12% in these Indian studies [5, 18, 24]. Neck pain or radicular symptoms were not seen and none had Horner's syndrome or sensory impairment in any of these studies [5, 18, 24].

The clinical findings reported by Hassan et al. [5] were more elaborate with greater proportion of patients showing the characteristic abnormalities of HD than those reported by Sonwalkar et al. [18] and Raval et al. [24] perhaps due to greater emphasis placed on the pattern of clinical involvement and its correlation with electrophysiology and dynamic MRI of cervical spine. These recent Indian studies not only reflect the heightened awareness of the disease amongst neurologists but may also suggest that specific environmental factors, ethnic background, or cultural and behavioural habits may be involved in the susceptibility to the disease [25].

## 3. Pathophysiology

Although Hirayama et al. first reported this disease in 1959, pathologic study of the affected segment of the spinal cord was not possible until 1982 when one of his patients after 23 years of followup had died of carcinoma lung at age of 38 years and was subjected to autopsy; detailed neuropathological findings were reported in 1987 [11, 16]. At pathologic examination, shrinkage, necrosis, and gliosis were found in the anterior horns of the spinal cord from C-5 to T-1, particularly marked at C-7 and C-8 [16]. The underlying pathogenesis of the disease was not known until 1987 when Kikuchi et al. first proposed tight dural canal as the underlying predisposing factor [26]. Atopy and elevated serum IgE level have also been postulated to be precipitating factors in HD [27, 28]. Benign monomelic amyotrophy had been considered variant of spinal muscular atrophy with focal emphasis and a benign course. Di Guglielmo et al. did not find deletions at the survival motor neuron (SMN) locus in benign monomelic amyotrophy of upper and lower limbs suggesting that these disorders are not only clinically but also genetically separate entities from proximal spinal muscular atrophies [29]. Misra et al. found that all patients were negative for SMN1 and SMN2 gene deletions in their study of fifteen patients of HD; exclusive occurrence in male patients and the presence of this disease in two brothers in the study suggested a possible role of X chromosome [30].

Notwithstanding these reports, the debate has continued whether this condition represents a focal form of primary lower motor neuron degeneration or a local consequence of anatomical variation in the cervical spine [31]. An imbalanced growth resulting in disproportional length between the patient's vertebral column and spinal canal contents has been suggested as the cause of a tight dural sac and anterior displacement of posterior dural wall on neck flexion [14, 15, 26, 32]. The different growth rates between males and females have been proposed, by Toma and Shiozawa, to be the factor related to the male preponderance of HD [33]. The disproportionate shortening of the dural sac is perhaps accentuated during the juvenile growth spurt, explaining the preponderance in adolescence [33].

In normal spine, the spinal dura mater is a loose sheath consisting of several transverse folds that is anchored in the vertebral canal by nerve roots and by its attachment to periosteum at foramen magnum and dorsal surfaces of C2-C3 vertebrae above and at coccyx below, which compensates for the increased length of the cervical canal in flexion [3, 12, 16]. The difference in length between extension and flexion from T-1 to top of atlas is 1.5 cm at the anterior wall and 5 cm at the posterior wall [34]. In health, the dura remains in close contact with the walls of the spinal canal without anterior displacement in flexion [3].

However, in patients with HD, on neck flexion the tight dural sac cannot compensate for the increased length of posterior wall, which causes anterior shifting of the posterior cervical dural wall and consequent compression of the cord against the posterior margin of adjacent vertebral bodies. This compression may cause microcirculatory disturbances in the territory of anterior spinal artery in the lower cervical spinal cord [12, 16]. The chronic circulatory disturbance resulting from repeated or sustained flexion of the neck may produce necrosis of the anterior horns resulting in gliosis and localized cord atrophy at the lower cervical region [7, 12], which is consistent with the pathologic finding on autopsy study [16]. Vulnerability of the anterior horns to ischemia accounts for the atrophy that follows [12]. Patients with severe cervical cord compression in flexion may also develop extensive cord injury beyond the anterior horns [35]. Patients with brisk deep tendon jerks likely have cord injury beyond the anterior horns [5].

## 4. Electrophysiology

Nerve conduction studies reveal variably reduced median and ulnar compound muscle action potentials [5, 18, 24]. Motor and sensory conduction velocities are normal in median and ulnar nerves [5, 18, 24]. However, distal latencies and F-wave latencies are either normal or variably affected [5, 18]. F waves are used to evaluate the proximal nerve conduction and anterior horn cell disorders. Because of chronic denervation there may be prolongation of latency, decline in persistence, and reduction in amplitude; however depending on reinnervation, the amplitudes may increase. In patients with severe wasting, the F wave may become unrecordable [36].

Restuccia et al. in a study of upper limb somatosensory evoked potentials demonstrated that neck flexion caused a significant amplitude decrease of the N13 cervical response in patients with HD but not in patients with amyotrophic lateral sclerosis (ALS) and healthy subjects, suggesting that direct cord compression or microvascular changes could in theory account for this position-related dysfunction [10]. However, Misra et al. found no significant change in somatosensory evoked potentials and F-wave parameters on neck flexion compared with neutral position in their study of eight patients of HD and controls [36]. Ammendola et al., in a small cohort of three cases, did not show statistically significant differences for F-wave, somatosensory evoked potentials and motor evoked potentials in standard conditions and during neck flexion both in HD patients and controls, suggesting that some cases of a complex disorder like HD might have a pathogenetic mechanism different from “flexion myelopathy” [37]. Misra and Kalita, in another study of seven patients of HD, found that the central motor conduction to lower limbs was normal in all while central motor conduction to upper limbs was marginally prolonged on one side [38]. 

Electromyography reveals features of active denervation in form of fibrillations, positive sharp waves, and fasciculations and features of chronic denervation in form of neurogenic changes in C7, C8, and T1 myotomes [5, 18, 24, 30]. Hassan et al. excluded upper limb involvement by demonstrating normal electromyography of C5, C6 myotomes, namely, deltoid, biceps brachii and brachioradialis (Figures 1(b), 1(c), and 1(d)) [5]. Similar sparing of C5, C6 myotomes has been reported by Ghosh et al. [9]. Cervical paraspinal electromyography is normal [18]. In an earlier study, Gourie-Devi et al. found the electromyographic features along with histologic features of neurogenic atrophy suggestive of anterior horn cell lesion [4].

## 5. Imaging

In patients with HD, conventional radiographic studies of the cervical spine may show loss of cervical lordosis [32]. Myelography is difficult to be performed as it is not easy to retain the contrast medium in cervical subarachnoid space when the neck is flexed [3]. MRI with flexion contrast study is the gold standard of diagnosis which should be done if the routine MRI otherwise looks normal and if MND has been excluded [33].

### 5.1. MRI Protocol

The optimal MRI protocol should include imaging in neutral position followed by imaging in hyperflexion. The sequences performed in neutral position include sagittal SE T1W, TSE T2, gradient echo T2, MR myelography in sagittal, and coronal planes. The sequences performed in hyperflexion of cervical spine include sagittal SE T1W with and without fat saturation, TSE T2, gradient echo T2, MR myelography in sagittal, and coronal planes. Postcontrast SE T1W is done in transverse plane. Maximal possible hyperflexion of neck is achieved by asking the subject to first move the head as forward as possible and then to touch the chin to the chest. The shoulders are pushed as far caudal as possible. The position is maintained by supporting the neck and shoulders with MR compatible foam pads. The gadolinium based MR contrast agent is used in the dose of 0.5 mmol/kg administered as bolus intravenously [5].

The following features are evaluated: (a) localized lower cervical cord atrophy, (b) asymmetric cord flattening, (c) abnormal cervical curvature, (d) loss of attachment between the posterior dural sac and subjacent lamina, (e) anterior shifting of the posterior wall of the cervical dural canal, (f) enhancing epidural component with flow voids, and (g) intramedullary signal hyperintensity [5]. The foregoing features are further elucidated below based on the definitions mentioned in the literature.

Lower cervical cord is defined as the cord between C4 and C7. Localized cord atrophy is defined as a decrease in cord size in comparison with the normal cord above and that below the affected level on sagittal MR images and confirmed on transverse MR images [14]. Asymmetric cord flattening is evaluated on transverse MR images. Cord flattening is defined as flattening without a narrowed or obliterated adjacent subarachnoid space. An elliptic spinal cord is considered normal, a pear-shaped spinal cord is considered asymmetric cord flattening, and a triangular spinal cord is considered symmetric cord flattening [19].

Cervical curvature is measured according to the relationship of the dorsal aspect of the vertebral bodies C3 through C6 (line A) to a line drawn from the dorsocaudal aspect of the body of C2 vertebra to the dorsocaudal aspect of the body of C7 vertebra (line B). By definition, normal lordotic cervical curvature is curvature in which no part of line A crosses the line B. An abnormal (straight or kyphotic) curvature is curvature in which part or all of line A meets or crosses through the line B [39].

For evaluating the loss of attachment between the posterior dural sac and subjacent lamina, the lamina is defined as the part of vertebra between junctions of laminae medially and laterally by a tangential line along the medial aspect of the pedicle. This is divided equally into three parts. More than one-third loss of attachment between the posterior dural sac and subjacent lamina is considered significant [19].

On dynamic flexion studies, anterior displacement of dural sac and appearance of epidural flow voids with enhancing epidural component posterior to the thecal sac are noted. Noncompressive intramedullary high signal intensity is considered if patent subarachnoid space and intramedullary high signal intensity are noted [19].

### 5.2. MRI Findings

The MRI findings reported more frequently from Japan and China as also from Europe and North America are asymmetrical or symmetrical atrophy of lower cervical cord, anterior shifting of posterior dural sac on flexion, and prominence and enhancement of posterior epidural venous plexus on dynamic flexion studies. Studies from these countries have reported loss of attachment between the posterior dural sac and subjacent lamina on neutral position and anterior shifting of the posterior wall of cervical dural canal, enhancing epidural crescentic mass in the lower cervical and thoracic region and prominent posterior epidural flow voids indicative of dilated epidural venous plexus on flexion studies as highly suggestive for the diagnosis of HD [3, 7, 12, 19]. Forward motion of posterior dura mater on MRI were described by Pradhan and Gupta in 1997 [14] and by Hirayama and Tokumaru in 2000 [15]. Pradhan and Gupta elaborately demonstrated forward displacement and flattening of cord against bodies of lower cervical vertebrae in flexed neck position [22]. 

**Figure 2:**
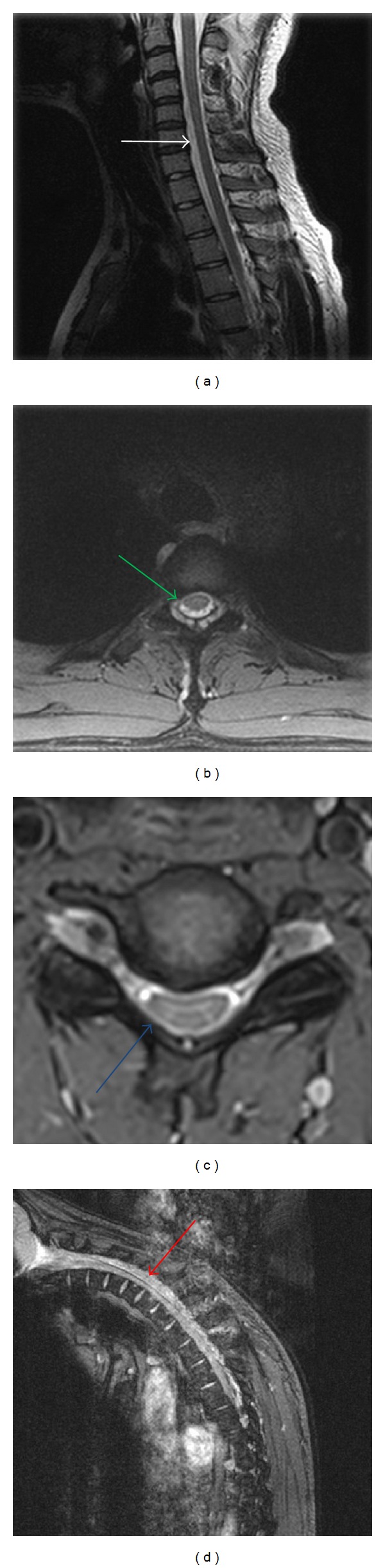
Localised cord atrophy (white arrow) on neutral sagittal T2WI (a), pear shaped asymmetric cord flattening on neutral transverse T2WI (green arrow) (b), asymmetric cord flattening with loss of attachment of dura from subjacent lamina (blue arrow) on neutral position axial T2WI MRI (c), and anterior displacement of posterior dura from C3–D8 on postcontrast imaging showing intense enhancement of posterior epidural space from C6–D3 (red arrow) (d).

Three recent series from India (Table 2) found a trend similar to the findings from other Asian countries and the west. Loss of cervical lordosis was found by Hassan et al. in 91% cases of HD, while Sonwalkar et al. found it in 75% and Raval et al. in 100% of their cases [5, 18, 24]. Lower cervical cord atrophy was found by Hassan et al. in 82% cases (Figure 2(a)), while Sonwalkar et al. and Raval et al. observed these findings in 100% of their cases [5, 18, 24]. Asymmetric cord flattening was reported by Hassan et al. (Figures 2(b) and 2(c)) and Raval et al. in 100% of their cases, while Sonwalkar et al. observed these findings in 75% of cases [5, 18, 24]. Loss of dural attachment with subjacent lamina was reported by Hassan et al. in 90% cases, while Sonwalkar et al. and Raval et al. reported these findings in 50% and 100% of their cases, respectively [5, 18, 24]. Anterior displacement of dorsal dura on neck flexion was observed by Hassan et al. in 90% cases, while Sonwalkar et al. and Raval et al. observed these findings in 75% and 100% of cases, respectively [5, 18, 24]. Prominent epidural flow voids were found by Hassan et al. in 90% cases, while Sonwalkar et al. and Raval et al. found these findings in 50% and 44% of their cases, respectively [5, 18, 24]. Enhancing epidural crescent in flexion was noted by Hassan et al. (Figure 2(d))and Raval et al. in 100% of their cases, while Sonwalkar et al. noted these findings in 75% of cases [5, 18, 24]. Intramedullary hyperintensity was reported by Hassan et al. in 18% cases, while Sonwalkar et al. and Raval et al. reported these findings in 37% and 44% of cases, respectively [5, 18, 24].

Among the imaging features discussed above, localized lower cervical cord atrophy, asymmetric cord flattening, and loss of dural attachment have an accuracy of 80% in identification of the disease [19, 20]. Chen et al. have proposed loss of attachment of cervical dura to subjacent lamina as the most valuable finding for diagnosing HD disease in the neutral position [19]. In the study by Hassan et al., localised lower cervical cord atrophy and loss of cervical lordosis were seen in over 80% cases of HD [5]. Tashiro et al. found focal cord atrophy in neutral neck position on MRI in 100% cases [13], while Hirayama and Tokumaru have reported this in only 50% of their cases [15]. Hassan et al. found asymmetric cord flattening and enhancing epidural crescent in flexion in all their cases, while loss of dural attachment, anterior displacement of dorsal dura, and epidural flow voids on neck flexion were seen in 90% cases [5]. The MRI findings in the series of Hassan et al. [5] were in greater conformity with those of Raval et al. [24] than Sonwalkar et al. [18] perhaps due to difference in patient characteristics and extent of disease.

Compression of spinal cord by tight dura is probably the most important pathogenetic factor. Hirayama in their study on 73 patients found that dynamic compression of the lower cervical cord due to forward displacement of the posterior cervical dural sac and spinal cord on neck flexion was confined to an early and progressive stage of the disease [1]. However, Pradhan and Gupta from their study on 35 patients showed that both spinal cord and posterior cervical dura move forward independently under a longitudinal stretch and that the forward displacement of the dura was not responsible for the cord compression [22]. 

Contrariwise, Lai et al. in their study found forward shifting of posterior cervical dura without associated cord compression in 46% of normal subjects upon flexion; the intrinsic compensatory mechanism is perhaps inadequate in patients of HD leading to cord compression. The authors concluded that depicting of forward shifting of posterior dura alone on flexion cannot reliably diagnose HD [40]. Intriguingly, Pradhan and Gupta have observed forward movement of the cervical cord even in normal individuals during flexion [22]. However, absence of forward displacement of dural sac in a later and nonprogressive stage of the disease suggests that the dynamic compression has pathogenic significance [1]. Interestingly, Hassan et al. observed that late-onset HD may have demonstrable forward dural shift in conformity with clinically progressive stage of the disease [5].

Venous congestion in flexion might also play an additional role in determining spinal cord ischemic changes [8]. The proposed mechanisms for venous engorgement seen on flexion studies are due to increased flow to posterior internal vertebral venous plexus resulting from the negative pressure in posterior spinal canal as a result of anterior shifting of dural canal and decrease in drainage of jugular veins impeding venous return of internal vertebral venous plexus [20]. Additionally, the compressed anterior internal vertebral venous plexus caused by anterior displacement of dural canal increases the burden of posterior internal vertebral venous plexus leading to its distension [3, 20]. Pradhan and Gupta have postulated that the overstretched posterior dura mater that forcefully moves forward during neck flexion results in negative space behind, which appears on MRI as congested epidural space; posterior dura mater came forward in all their patients resulting in a dilated crescent-shaped epidural space [22]. Even in bilaterally symmetric form of HD, Pradhan demonstrated on flexion MRI severe flattening of lower cervical cord against C5-C6 vertebrae and development of crescent-shaped enhancing epidural space extending from C4 to T2 spine [23], which has been corroborated by other Indian studies [5, 18, 24].

Hassan et al. demonstrated that while the clinical-electrophysiological profile was suggestive of anterior horn cell disease, dynamic flexion MRI of the cervical spine supported the theory of forward dural shift causing lower cervical cord compression and ischemia of anterior horns. Their study supports the hypothesis that HD differs from classic MND or its variants involving the distal upper limbs in young by being a chronic ischemic myelopathy rather than a degenerative condition [5]. 

Though a diagnosis of Hirayama disease is straightforward at flexion MR imaging, the challenge for radiologists is how to identify this condition on routine nonflexion MR studies [3]. To ensure that this diagnosis is not missed in patients presenting with focal hand wasting, flexion contrast MRI should be done if the routine MRI otherwise looks normal and if motor neuron disease has been excluded. Several conditions like syringomyelia, motor neuron disease, cervical spondylotic myelopathy, spinal cord tumor, and traumatic myelopathy may cause localized amyotrophy of the distal arm, and these should be excluded first by imaging modalities [33]. HD should also be considered in the differential diagnosis of multifocal motor neuropathy and juvenile form of ALS [41].

**Figure 3:**
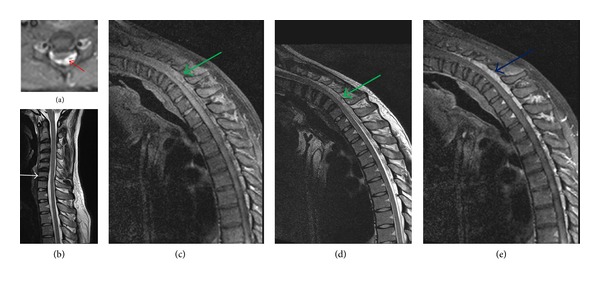
A 23-year-male with weakness and wasting of left hand and forearm with anteroposterior pear-shaped cord flattening with loss of attachment of dura from subjacent lamina (red arrow) on T2WI axial MRI (a), midsagittal image showing loss of cervical lordosis (white arrow) but not showing cervical cord atrophy on neutral position sagittal T2WI MRI (b), dorsal epidural voids (green arrows) on flexion sagittal T1WI and T2WI MRI ((c), (d)), and posterior epidural crescentic enhancing mass (blue arrow) on flexion contrast sagittal T1WI MRI (e).

There is paucity of data on well-controlled studies of MR findings in HD [22, 40]. More elaborate case control studies on the subject are needed to conclusively identify the hallmark radiological features of the disease. Until more information is made available, we feel that asymmetric cord flattening and loss of dural attachment on transverse neutral MRI are the most important clue to diagnosis of HD; anterior displacement of dura and enhancing posterior epidural component have high predictivity for the diagnosis of HD. Typical MRI findings in a classic HD is shown in Figure 3. Need for dedicated neuroimaging of the cervical spine in young adolescents with hand wasting cannot be overemphasized as early institution of cervical collar therapy may be beneficial in patients of HD.

## 6. Nosologic Position: Monomelic Amyotrophy or Hirayama Disease?

Gourie-Devi et al. in 1984 first proposed the term “monomelic amyotrophy” since the striking feature in this condition was atrophy of one limb. They believed that as one upper or lower limb was involved, this nomenclature appeared more appropriate than the label juvenile muscular atrophy of unilateral upper extremity suggested by Hirayama et al. in 1959 [4, 11]. However, we feel neither nomenclature is appropriate. 

Since then MMA has been traditionally taught as a variant of MND and has been subclassified under idiopathic group of MND seen in India [42]. Subsequently, young patients with unilateral upper limb atrophy have been classically labeled “brachial MMA” or simply “MMA,” which in fact represent cases of HD. The clinical description of patients with MMA reported by Gourie-Devi et al. is similar to the clinical profile of HD described by Hirayama [1, 4]. 

Interestingly, Hirayama's original description of juvenile muscular atrophy of “unilateral” upper extremity is itself not in keeping with the more common bilateral asymmetric disease. Cases described by Hassan et al. are closer to the classic description of HD as we know today since these authors included both unilateral and bilateral asymmetric distal upper limb wasting and excluded patients with proximal upper limb wasting [1, 5], while Gourie-Devi et al. included patients with strictly unilateral upper limb atrophy, both distal and proximal involvements [4]. 

Pradhan, in their series of 106 patients of HD seen from 1992 to 2008, reported around 10% of all their patients to have bilaterally symmetric involvement, a severe form of classic HD which remains undiagnosed due to a common notion that it is a unilateral or grossly asymmetric disease [23]. We agree with Pradhan that the cases of MMA represent HD [23]. Gourie-Devi and Nalini found involvement of contralateral upper limb in up to 20% of forty-four patients followed up over mean of nearly ten years [21]. Ghosh et al. have shown evidence of bilateral electrophysiologic changes even in face of unilateral clinical involvement [9]. 

**Figure 4:**
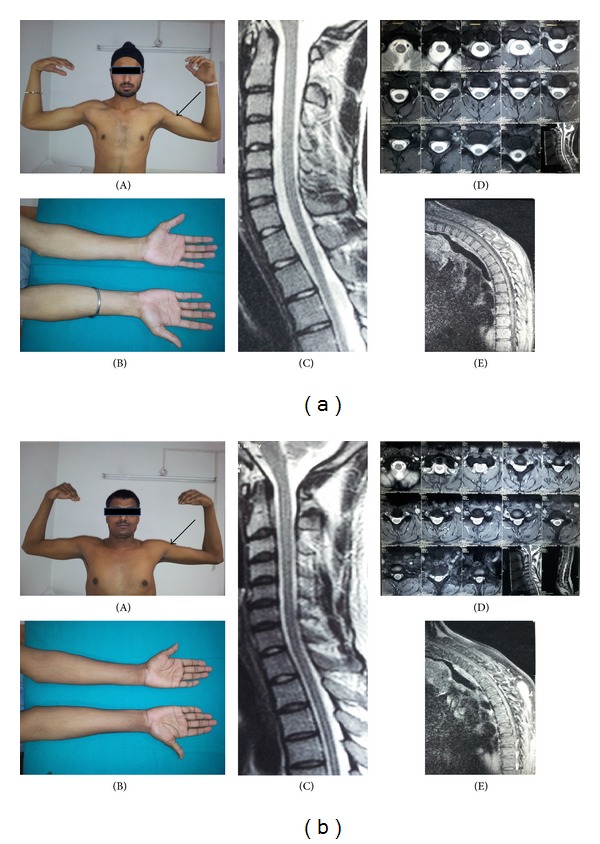
Two young males (a) and (b) with proximal left upper limb weakness and wasting without the typical findings described in Figures 1 to 3: marked wasting proximal left upper limbs (black arrow) (A) and mild/no wasting distal left upper limbs in same patients (B), preserved or mild loss of cervical lordosis and absence of localized cord atrophy at corresponding C5, 6 segments (C), normal cervical cord outline without loss of attachment of dura from subjacent lamina at all levels on neutral position axial T2WI MRI (D), and no posterior epidural crescentic enhancing mass on flexion contrast sagittal T1WI MRI (E).

Bilateral involvement, as noted by several authors including Gourie-Devi as also our own experience, calls for review of the nomenclature “monomelic amyotrophy” described to denote this disease. As dynamic compression has been classically demonstrated in lower cervical segments it would be interesting to subject cases of classic MMA with proximal upper limb atrophy to dynamic MRI studies; absence of dynamic compression in such cases may call for exploring other etiologies as cause of “proximal” MMA. As such there is need to exclude or remove the many nomenclatures of this not so uncommon entity and “lump” them under the rubric of “Hirayama disease.” In our view the term MMA may be restricted to cases of proximal upper limb atrophy which do not show typical MR features of HD (Figures 4(a) and 4(b)). This subset of patients may have a pathogenetic basis different from the dynamic ischemic changes of lower cervical cord postulated for distal limb wasting of Hirayama disease.

## 7. Treatment

### 7.1. Collar Therapy

Application of a cervical collar to minimize neck flexion prevents progressive muscular weakness in the early stages of the disease. The value of cervical collar use in HD is substantiated by electrophysiological studies showing further prolongation of latency and impersistence of F wave and/or increased latency and decreased amplitude of motor evoked potentials on neck flexion. These phenomena should be indicators to start and/or stop cervical collar therapy [43].

HD patients have an increased range of cervical spine flexion which contributes to the pathophysiological changes [44]. Though HD is self-limiting, early diagnosis and therapeutic intervention in form of cervical collar therapy to prevent neck flexion may minimize the functional disability in young patients [1, 3, 5, 45]. Cervical collar therapy induces a premature arrest of this disease. Improvement is expected in patients who have shorter duration of illness and have mild cord atrophy in neutral neck position [45].

Tokumaru and Hirayama in 1992 reported the use of cervical collar therapy for nonprogressive juvenile spinal muscular atrophy of the distal upper limb [46]. Pradhan and Gupta in 2001 recommended cervical collar during the acute progressive stage of the disease [22]. This was followed by a report by Tokumaru and Hirayama in 2001 on role of cervical collar therapy in 38 cases of HD [45]. They found that all patients on treatment showed no further progression after introduction of cervical collar. In the study by Hassan et al., five patients who were compliant with cervical collar use had subjective feeling of arrest of disease progression after 2 to 3 months, whilst six others without collar use felt slowing of progression only after 12 to 18 months of presentation [5]. Since the progressive stage is expected to cease in a few years, application of a cervical collar for three to four years generally has been advocated [45]. 

### 7.2. Role of Surgery

While application of cervical collar is believed to prevent progression of the disease in early stages, duraplasty, anterior cervical decompression, and reconstructions with tendon transfers have yielded encouraging results in selected patients [43, 45, 47]. Surgery seems to be beneficial for patients who do not respond to conservative treatment for more than 5 years after their onset, as this gives permanent stable fixation with much shorter period of external cervical immobilization compared with cervical collar therapy in which long-term application is frequently unbearable in many patients [48]. Konno et al. proposed the usefulness of cervical duraplasty with posterior spinal fusion in patients with dynamic cord compression and dural shift who show no improvement with cervical collar for more than 3 months [49]. Imamura et al. reported a 16-year-old boy with classic HD with remarkable improvement of muscle strength following an anterior C5 vertebrectomy followed by fixation of C4–6 vertebral bodies using iliac bone and plate system [48]. Chiba et al. reported tendon transfer as an operative reconstruction to improve the intricate finger movements in advanced HD [43].

Lack of good quality controlled trials of either collar therapy or surgical interventions makes drawing firm conclusions of any benefit from these therapeutic options fraught with probability of subjectivity and reporting of treatment successes heavily biased by outcome. Controlled clinical trials on therapy of HD are required to establish their role in clinical practice.

## 8. Conclusion

Hirayama disease, a rare disease affecting young men in the second to third decades of life, is characterized by insidious onset and slowly progressive course followed few years later by static phase of unilateral or asymmetric atrophy of the hand(s) and forearm(s) with sparing of the brachioradialis characterized as oblique amyotrophy. It is thought to be an ischemic cervical flexion myelopathy related to sustained or repeated movements of the neck causing chronic microcirculatory changes in the territory of anterior spinal artery supplying the anterior horns of lower cervical cord. While dynamic contrast MRI is very supportive of the diagnosis of Hirayama disease, routine MRI has high predictive value for the diagnosis. Prompt clinical diagnosis of Hirayama disease with supportive information from EMG/NCS and imaging is important to institute early cervical collar therapy in young adolescents with focal upper limb wasting.

Since the first description of monomelic amyotrophy by Gourie-Devi et al. in 1984, subsequent case series on juvenile muscular atrophy of upper extremity by Pradhan et al., Misra et al., and Hassan et al. from India have found bilateral involvement as a rule rather than an exception. Gourie-Devi's series of monomelic amyotrophy included both proximal and distal limb involvements and some of these later developed bilateral diseases. The clinical description of patients reported by all these authors is similar to the clinical profile of Hirayama disease described by Hirayama [1, 4]. Moreover, demonstration, by several workers, of lower cervical dynamic compression on MRI in juvenile muscular atrophy of upper extremity is not in agreement with the traditional classification of monomelic amyotrophy as a variant of motor neuron disease. We propose a common rubric of “Hirayama disease” for all such cases restricting the term monomelic amyotrophy for cases of proximal upper limb atrophy which do not show typical MR features of Hirayama disease. Till controlled trials on the various treatment options are available cervical collar therapy is recommended in Hirayama disease.

## Figures and Tables

**Table 1:** Clinical profile of Hirayama disease [4, 5, 18, 24].

Clinical feature	Hassan et al. [5] (2012): no. (%)	Sonwalkar et al. [18] (2008): no. (%)	Raval et al. [24] (2010): no. (%)	Gourie-Devi et al. [4] (1984): no. (%)
Total number of patients	11	8	9	13
Hand wasting	11 (100)	6 (75)	*	12 (92)
Forearm wasting	11 (100)	4 (50)	*	13 (100)
Brachioradialis sparing (oblique amyotrophy)	11 (100)	3 (37)	6 (67.5)	11 (85)
Proximal (C5,6) myotomal wasting	- (was exclusion criteria)	1 (12)	—	9 (69)**
Hand weakness	11 (100)	8 (100)	*	12 (92)
Forearm weakness	8 (73)	6 (75)	*	13 (100)
Minipolymyoclonus	10 (91)	5 (62)	5 (55)	11 (85)
Brisk deep tendon reflexes	2 (18)	1 (12)	—	Nil
Bilateral involvement	6 (55)	6 (75)	1 (11)	- (was exclusion criteria)
Unilateral involvement	5 (45)	2 (25)	8 (89)	13 (100)
Cold paresis	4 (36)	—	5 (55)	6 (46)
Fasciculations	3 (27)	—	5 (55)	6 (46)
Dysaesthesia on hand	1 (9)	1 (12)	1 (11)	1 (8)

*Mentioned as weakness and wasting in upper limb(s): right 4, left 4, and bilateral 1.

**Scapular 3, pectoralis 2, deltoid 5, and biceps 5.

**Table 2:** MRI findings in Hirayama disease [5, 18, 24].

MRI feature	Hassan et al. [5] (2012): no. (%)	Sonwalkar et al. [18] (2008): no. (%)	Raval et al. [24] (2010): no. (%)
*Total number of patients *	11*	**8**	**9**
Neutral position			
Abnormal cervical curvature (loss of cervical lordosis)	10/11 (91)	6 (75)	9 (100)
Localised lower cervical cord atrophy	9/11 (82)	8 (100)	9 (100)
Asymmetric cord flattening	11/11 (100)	6 (75)	9 (100)
Intramedullary hyperintensity in lower cervical cord	2 (18)	3 (37)	4 (44)
Flexion position			
Loss of attachment between posterior dural sac and subjacent lamina	9/10 (90)	4 (50)	9 (100)
Anterior shifting of posterior cervical dural wall on flexion	9/10 (90)	6 (75)	9 (100)
Prominent epidural flow voids	9/10 (90)	4 (50)	4 (44)
Enhancing epidural mass in lower cervical region	10/10 (100)	6 (75)	9 (100)

*11 patients underwent routine neutral position MRI, and 10 underwent flexion contrast MRI.

## References

[1] Hirayama K (2008). Juvenile muscular atrophy of unilateral upper extremity (Hirayama disease)—half-century progress and establishment since its discovery. *Brain and Nerve*.

[2] Kikuchi S, Tashiro K, Kitagawa M, Iwasaki Y, Abe H (1987). A mechanism of juvenile muscular atrophy localized in the hand and forearm (Hirayama’s disease)—flexion myelopathy with tight dural canal in flexion. *Clinical Neurology*.

[3] Chen C, Chen C, Wu C, Ro L, Chen S, Lee T (1998). Hirayama disease: MR diagnosis. *American Journal of Neuroradiology*.

[4] Gourie-Devi M, Suresh TG, Shankar SK (1984). Monomelic amyotrophy. *Archives of Neurology*.

[5] Hassan KM, Sahni H, Jha A (2012). Clinical and radiological profile of Hirayama disease: a flexion myelopathy due to tight cervical dural canal amenable to collar therapy. *Annals of Indian Academy of Neurology*.

[6] Biondi A, Dormont D, Weitzner I, Bouche P, Chaine P, Bories J (1989). MR imaging of the cervical cord in juvenile amyotrophy of distal upper extremity. *American Journal of Neuroradiology*.

[7] Schröder R, Keller E, Flacke S (1999). MRI findings in Hirayama’s disease: flexion-induced cervical myelopathy or intrinsic motor neuron disease?. *Journal of Neurology*.

[8] Ciceri EF, Chiapparini L, Erbetta A (2010). Angiographically proven cervical venous engorgement: a possible concurrent cause in the pathophysiology of Hirayama’s myelopathy. *Neurological Sciences*.

[9] Ghosh PS, Moodley M, Friedman NR, Rothner AD, Ghosh D (2011). Hirayama disease in children from North America. *Journal of Child Neurology*.

[10] Restuccia D, Rubino M, Valeriani M, Mirabella M, Sabatelli M, Tonali P (2003). Cervical cord dysfunction during neck flexion in Hirayama’s disease. *Neurology*.

[11] Hirayama K, Toyokura Y, Tsubaki T (1959). Juvenile muscular atrophy of unilateral upper extremity: a new clinical entity. *The Japanese Journal of Psychiatry and Neurology*.

[12] Hirayama K, De Jong JM (1991). Non-progressive juvenile spinal muscular atrophy of the distal upper limb [Hirayama's disease]. *Handbook of Clinical Neurology*.

[13] Tashiro K, Kikuchi S, Itoyama Y (2006). Nationwide survey of juvenile muscular atrophy of distal upper extremity (Hirayama disease) in Japan. *Amyotrophic Lateral Sclerosis*.

[14] Pradhan S, Gupta RK (1997). Magnetic resonance imaging in juvenile asymmetric segmental spinal muscular atrophy. *Journal of the Neurological Sciences*.

[15] Hirayama K, Tokumaru Y (2000). Cervical dural sac and spinal cord in juvenile muscular atrophy of distal upper extremity. *Neurology*.

[16] Hirayama K, Tomonaga M, Kitano K (1987). Focal cervical poliopathy causing juvenile muscular atrophy of distal upper extremity: a pathological study. *Journal of Neurology Neurosurgery and Psychiatry*.

[17] Tokumaru Y, Hirayama K (1989). Anterior shift of posterior lower cervical dura mater in patients with juvenile muscular atrophy of unilateral upper extremity. *Clinical Neurology*.

[18] Sonwalkar H, Shah R, Khan F (2008). Imaging features in Hirayama disease. *Neurology India*.

[19] Chen C, Hsu H, Tseng Y (2004). Hirayama flexion myelopathy: neutral-position MR imaging findings—importance of loss of attachment. *Radiology*.

[20] Mukai E, Matsuo T, Muto T, Takahashi A, Sobue I (1987). Magnetic resonance imaging of juvenile-type distal and segmental muscular atrophy of the upper extremities. *Clinical Neurology*.

[21] Gourie-Devi M, Nalini A (2003). Long-term follow-up of 44 patients with brachial monomelic amyotrophy. *Acta Neurologica Scandinavica*.

[22] Pradhan S, Gupta RK (2001). Cervical dural sac and spinal cord in juvenile muscular atrophy of distal upper extremity. *Neurology*.

[23] Pradhan S (2009). Bilaterally symmetric form of Hirayama disease. *Neurology*.

[24] Raval M, Kumari R, Dung Dung A, Guglani B, Gupta N, Gupta R (2010). MRI findings in Hirayama disease. *Indian Journal of Radiology and Imaging*.

[25] Fetoni V, Briem E, Carrara F, Mora M, Zeviani M (2004). Monomelic amyotrophy associated with the 7472insC mutation in the mtDNA tRNASer(UCN) gene. *Neuromuscular Disorders*.

[26] Kikuchi S, Tashiro K, Kitagawa M, Iwasaki Y, Abe H (1987). A mechanism of juvenile muscular atrophy localized in the hand and forearm (Hirayama’s disease)—flexion myelopathy with tight dural canal in flexion. *Clinical Neurology*.

[27] Kira J, Ochi H (2001). Juvenile muscular atrophy of the distal upper limb (Hirayama disease) associated with atopy. *Journal of Neurology Neurosurgery and Psychiatry*.

[28] Chen T, Hung C, Hsieh T, Lu S, Yang S, Jong Y (2010). Symmetric atrophy of bilateral distal upper extremities and hyperigeaemia in a male adolescent with hirayama disease. *Journal of Child Neurology*.

[29] Di Guglielmo G, Brahe C, Di Muzio A, Uncini A (1996). Benign monomelic amyotrophies of upper and lower limb are not associated to deletions of survival motor neuron gene. *Journal of the Neurological Sciences*.

[30] Misra UK, Kalita J, Mishra VN, Kesari A, Mittal B (2005). A clinical, magnetic resonance imaging, and survival motor neuron gene deletion study of Hirayama disease. *Archives of Neurology*.

[31] Talbot K (2004). Monomelic amyotrophy or Hirayama’s disease. *Practical Neurology*.

[32] Mukai E, Sobue I, Muto T (1985). Abnormal radiological findings on juvenile-type distal and segmental muscular atrophy of upper extremities. *Clinical Neurology*.

[33] Toma S, Shiozawa Z (1995). Amyotrophic cervical myelopathy in adolescence. *Journal of Neurology Neurosurgery and Psychiatry*.

[34] Bland JH, Bland JH (1994). Basic anatomy. *Disorders of the Cervical Spine: Diagnosis and Medical Management*.

[35] Sakai K, Ono K, Okamoto Y, Murakami H, Yamada M (2011). Cervical flexion myelopathy in a patient showing apparent long tract signs: a severe form of Hirayama disease. *Joint Bone Spine*.

[36] Misra UK, Kalita J, Mishra VN, Phadke RV, Hadique A (2006). Effect of neck flexion on F wave, somatosensory evoked potentials, and magnetic resonance imaging in Hirayama disease. *Journal of Neurology, Neurosurgery and Psychiatry*.

[37] Ammendola A, Gallo A, Iannaccone T, Tedeschi G (2008). Hirayama disease: three cases assessed by F wave, somatosensory and motor evoked potentials and magnetic resonance imaging not supporting flexion myelopathy. *Neurological Sciences*.

[38] Misra UK, Kalita J (1995). Central motor conduction in Hirayama disease. *Electroencephalography and Clinical Neurophysiology*.

[39] Batzdorf U, Batzdorff A, Sypert GW (1988). Analysis of cervical spine curvature in patients with cervical spondylosis. *Neurosurgery*.

[40] Lai V, Wong YC, Poon WL, Yuen MK, Fu YP, Wong OW (2011). Forward shifting of posterior dural sac during flexion cervical magnetic resonance imaging in Hirayama disease: an initial study on normal subjects compared to patients with Hirayama disease. *European Journal of Radiology*.

[41] Silani V, Messina S, Poletti B (2011). The diagnosis of Amyotrophic Lateral Sclerosis. *Archives Italiennes de Biologie*.

[42] Velmurugendran CU, Srinivasan AV, Chopra JS, Sawhney IMS (2004). Motor neuron disease in tropics. *Neurology in Tropics*.

[43] Chiba S, Yonekura K, Nonaka M, Imai T, Matumoto H, Wada T (2004). Advanced Hirayama disease with successful improvement of activities of daily living by operative reconstruction. *Internal Medicine*.

[44] Xu X, Han H, Gao H (2011). The increased range of cervical flexed motion detected by radiographs in Hirayama disease. *European Journal of Radiology*.

[45] Tokumaru Y, Hirayama K (2001). Cervical collar therapy for juvenile muscular atrophy of distal upper extremity (Hirayama disease): results from 38 cases. *Clinical Neurology*.

[46] Tokumaru Y, Hirayama K (1992). A cervical collar therapy for non-progressive juvenile spinal musclar atrophy in the distal upper limb (Hirayama’s disease). *Clinical Neurology*.

[47] Lin M, Kung W, Chiu W, Lyu R, Chen C, Chen T (2010). Hirayama disease: clinical article. *Journal of Neurosurgery*.

[48] Imamura H, Matsumoto S, Hayase M, Oda Y, Kikuchi H, Takano M (2001). A case of Hirayama’s disease successfully treated by anterior cervical decompression and fusion. *Brain and Nerve*.

[49] Konno S, Goto S, Murakami M, Mochizuki M, Motegi H, Moriya H (1997). Juvenile amyotrophy of the distal upper extremity: pathologic findings of the dura mater and surgical management. *Spine*.

